# Advances in Flotation Reagents for Cassiterite Separation: Challenges and Sustainable Solutions

**DOI:** 10.3390/molecules30112380

**Published:** 2025-05-29

**Authors:** Xianchen Wang, Hong Li, Xinhong Liu, Yuan Tang, Chenquan Ni

**Affiliations:** 1 School of Resources and Environmental Engineering, Moutai Institute, Renhuai 564507, China; lihong@mtxy.edu.cn (H.L.); liuxinhong@mtxy.edu.cn (X.L.); 2 Engineering Research Center of Phosphorus Resources Development and Utilization of Ministry of Education, Wuhan Institute of Technology, Wuhan 430073, China; yuan.tang@wit.edu.cn; 3 School of Resources and Safety Engineering, Wuhan Institute of Technology, Wuhan 430073, China; 4 School of Minerals Processing and Bioengineering, Central South University, Changsha 410083, China; 215601027@csu.edu.cn

**Keywords:** cassiterite, flotation reagent, collector, depressant, activator

## Abstract

Tin is a crucial strategic metal, extensively employed in aerospace, new energy materials, and other advanced fields. However, with the progressive depletion of high-grade tin ores, the utilization of low-grade tin ores for metal tin production has emerged as a significant trend. Nonetheless, low-grade tin ores present inherent challenges that hinder their direct application in tin extraction. Flotation remains an effective method to enhance ore grade, yet issues such as fine particle dispersion and ore complexity persist. In light of this, the present study provides a comprehensive review of cassiterite resource characteristics, surface chemistry, flotation reagents, and relevant case studies. By delving into the physicochemical properties of cassiterite, this paper elucidates its floatability and the distinctions among various flotation reagents. Furthermore, it identifies critical challenges in cassiterite flotation and proposes targeted, feasible strategies to support the efficient exploitation of tin resources, thereby fostering the sustainable development of the tin industry.

## 1. Introduction

**Figure 1:**
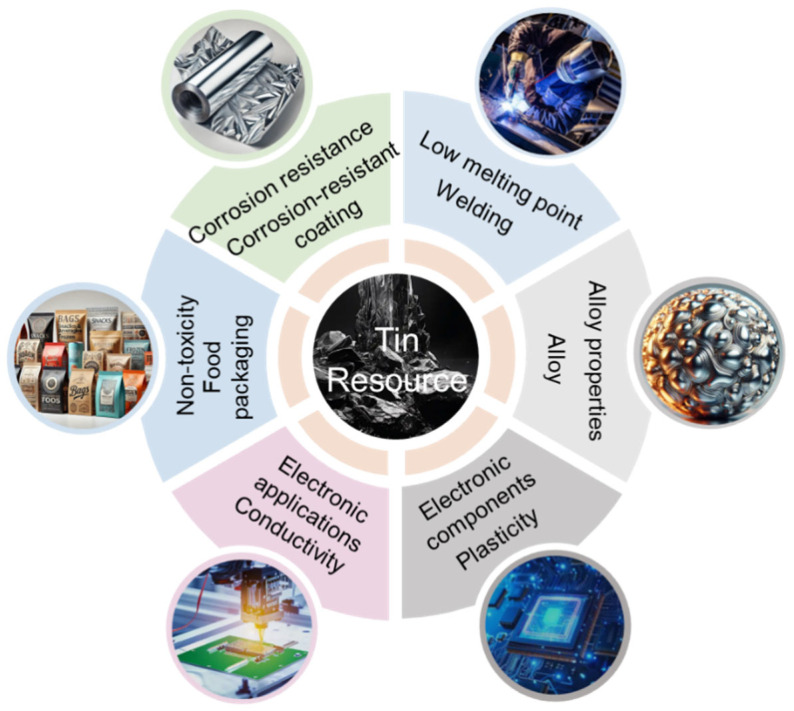
Applications of tin.

Tin (Sn) is indispensable in both industry and daily life, widely utilized in electronics, electrical appliances, information technology, construction materials, machinery, the automotive industry, aerospace, and nuclear energy due to its stable chemical properties, resistance to oxidation, low melting point, excellent ductility, and non-toxic nature (Figure 1) [1,2,3,4,5]. With the growing demand for Sn in emerging fields, the consumption of tin metal is steadily increasing [6,7,8]. However, the increasingly prominent bottleneck in resource supply severely restricts the development of the tin industry [9,10]. Currently, high-grade tin ores used for tin production have been largely depleted, and how to harness low-grade tin ores for production has become a pressing issue [11,12,13].

**Figure 2:**
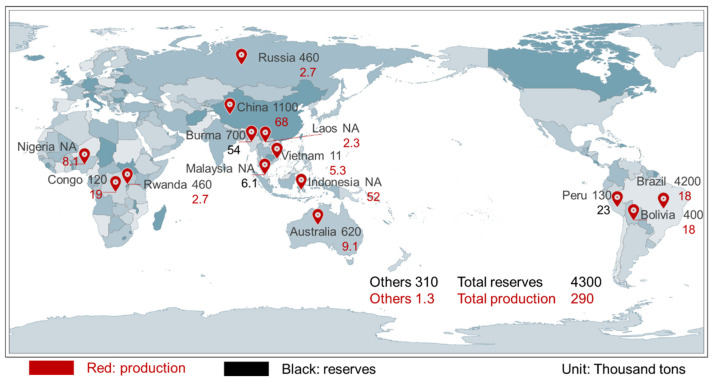
World cassiterite reserves and production in 2024 [14,15].

According to the data compilation from the United States Geological Survey (USGS), the global proven Sn reserves are estimated to be approximately 4.3 million tons in 2024, which primary located in Western Africa, Southeastern Asia, Australia, Bolivia, Brazil, Indonesia, and Russia [14,15]. The global production of Sn is approximately 290,000 tons, primarily in China, Indonesia, Myanmar, and Peru. Figure 2 illustrates the distribution of global Sn resources and the Sn ore production of the leading nations.

Over fifty types of tin-bearing minerals and ores have been discovered, with more than twenty being major minerals [16,17]. Common tin minerals include cassiterite, stannite, canfieldite, among others. The primary source of tin metal at present is cassiterite. Given its high density, chemical stability, and hardness, gravity separation is the foremost method for recovering cassiterite [11,18,19]. However, with the depletion of high-quality cassiterite resources, the extreme brittleness of tin ores results in generating a large volume of fine particles with small mass, large specific surface area, and high surface energy during the mining process. This renders traditional gravity separation methods ineffective, reducing resource utilization efficiency [20].

Flotation is the primary method for processing and utilizing complex ores [21,22,23,24]. This technique enhances the flotation differences between various minerals by modifying the surface interfaces of the minerals, thereby facilitating their separation and concentration [25,26]. With the advancement of beneficiation equipment, alongside the continuous development of flotation reagents and processing techniques, the recovery of fine cassiterite has become increasingly feasible [27]. During the flotation of cassiterite, it often exhibits similar flotation characteristics to gangue minerals such as quartz and fluorite, resulting in the persistent issue of low concentrate grade and recovery rates in the flotation of fine cassiterite [28,29,30]. Flotation reagents are chemical agents that facilitate solid–liquid separation and enhance flotation performance during the ore flotation. They can increase the contact area between the ore and air bubbles through mechanisms such as adsorption or activation [31,32,33]. By improving the flotation differences between cassiterite and gangue minerals, these reagents enable the selective separation of fine cassiterite, overcoming challenges in recovery and concentrate grade [34,35]. Therefore, improving the flotation efficiency of cassiterite has been a hot topic for decades. This review summarizes the similarities and differences of cassiterite flotation and the progress of corresponding flotation research to guide the efficient utilization of cassiterite resources in the future.

The key to successful flotation lies in the selection of appropriate flotation reagents. Based on this, the present study analyzes the surface characteristics of cassiterite concerning the types of existing collectors, activators, and depressants, exploring their mechanisms of action. This work aims to provide valuable insights for future research on flotation reagents.

## 2. Surface Chemistry and Crystalline Structure

### 2.1. Crystalline Structure

**Figure 3:**
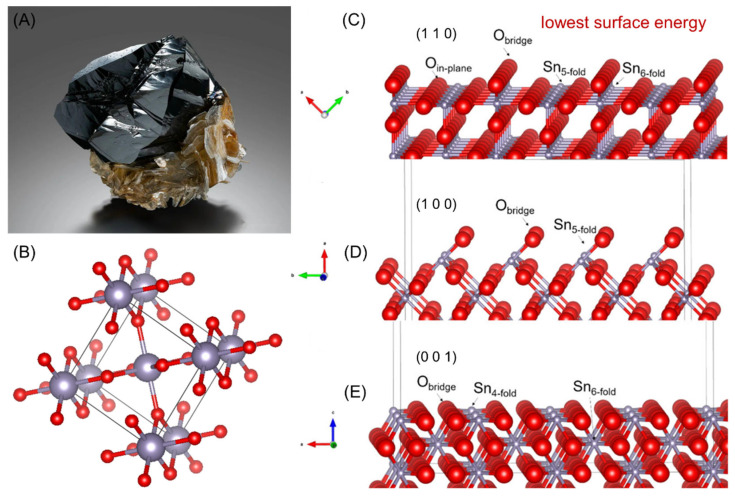
(**A**) Cassiterite image; (**B**) cassiterite crystal; (**C**) cassiterite (1 1 0) surface; (**D**) cassiterite (1 0 0) surface; (**E**) cassiterite (0 0 1) surface [40].

Cassiterite (SnO_2_) theoretically contains 78.6% tin and is a crystalline oxide mineral belonging to the tetragonal crystal system (as shown in Figure 3A). Cassiterite has a rutile-type structure and is typically found as short prismatic crystals with bipyramidal forms. Occasionally, it appears as elongated prisms or bipyramidal shapes [36]. The unit cell parameters are: a_0_ = b_0_ = 0.4738 nm, c_0_ = 0.3186 nm, with α = β = γ = 90°, and Z = 2. The crystal structure is depicted in Figure 3B. Cassiterite is a typical AX_2_-type compound with a crystal structure characterized by hexagonal close packing. Sn^2+^ occupy the octahedral voids, with each O^2−^ surrounded by three Sn^2+^. The coordination number of Sn^2+^ is 6, while the coordination number of O^2−^ is 8. Cassiterite is a typical oxide mineral with relatively weak hydrophobicity on its surface [37]. Under external force, minerals generally fracture along planes with larger interlayer spacing, planes where the ionic charge balance occurs, adjacent layers of ions, or directions of weakest chemical bonding. Studies have shown that the (1 1 0) face of cassiterite exhibits the greatest stability and the lowest surface energy (as shown in Figure 3C–E) [38,39,40].

### 2.2. Surface Chemistry

**Figure 4:**
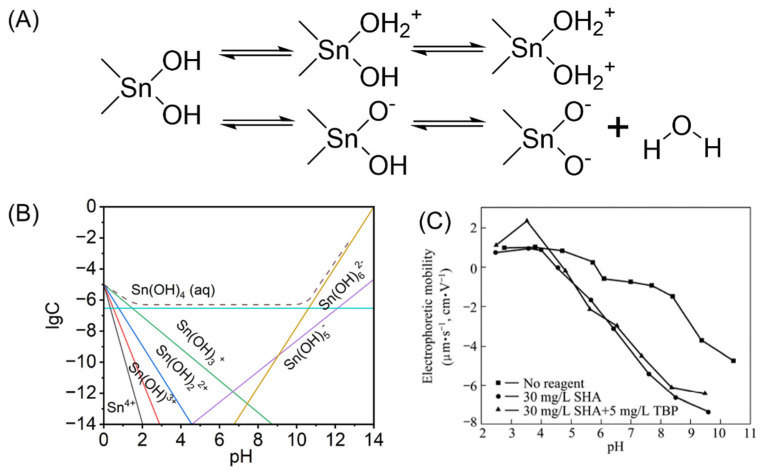
(**A**) Surface electrical model of cassiterite. (**B**) Dissolved components of cassiterite [41]. (**C**) Relationship between SHA and electrophoretic mobility, pH [50].

The surface of cassiterite particles consists of O^2−^ and Sn^4+^ ions arranged with unsaturated valence bonds, resulting in strong hydrophilicity. When wetted by aqueous solutions, it adsorbs water molecules and exhibits varying surface charges. This phenomenon can be represented by the surface charge model of cassiterite shown in Figure 4A. As shown in Figure 4B, at high H^+^ concentrations in the aqueous solution, cassiterite predominantly exists in the forms of Sn^4+^, Sn(OH)^3+^, Sn(OH)_2_^2+^, and Sn(OH)_3_^+^, resulting in a positively charged surface [41]. At high OH^−^ concentrations in the aqueous solution, cassiterite primarily exists in Sn(OH)_5_^−^ and Sn(OH)_6_^2−^, leading to a negatively charged surface. The electrical double layer largely governs the adsorption of flotation reagents. The most commonly used parameter is the zeta potential, particularly the isoelectric point (IEP). Changes in the IEP value can be used to measure the adsorption intensity of reagents on the mineral surface [42,43,44]. Table 1 summarizes the surface chemistry studies conducted on cassiterite, focusing primarily on the reported IEP [45,46]. The IEP value of cassiterite varies significantly, ranging from 2.9 to 7.3. This variability may be attributed to differences in potential-determining ions, mineral composition, surface structure, and the procedures and techniques used to determine the IEP [47,48,49]. Qin [50] reported an IEP for cassiterite samples at pH 5.8, which differs from the experimental results obtained by previous researchers (as shown in Figure 4C). The discrepancy arises from the adsorption of SO_4_^2−^ on the cassiterite surface, which influences its isoelectric point. The adsorption of anions on the surface facilitates the exchange adsorption of collectors. In the presence of salicylhydroxamic acid (SHA), the IEP of cassiterite shifts toward a more acidic pH, approximately 4.5. The effect of SHA on cassiterite causes its electrophoretic mobility to shift more negatively within the pH range of 4.5 to 10, indicating an increase in negative surface charge. This suggests that the collector overcomes electrostatic repulsion to adsorb onto the surface of cassiterite. Therefore, the interaction between reagents and cassiterite can influence the surface charge of the cassiterite.

## 3. Collectors

Naturally, hydrophobic minerals are rare. In flotation processes, the hydrophobicity of minerals is typically induced by the interaction between the mineral and the collector. In contrast, the hydrophobicity of non-target minerals is reduced by depressants [59,60,61,62]. Furthermore, activators play a pivotal role in flotation by modifying the surface properties of minerals, such as removing oxide layers or introducing active sites, thereby enhancing the interaction between the target mineral and the collector and improving its floatability. Thus, efficient and highly selective cassiterite collectors play a crucial role in cassiterite flotation and have been a significant research focus in fine cassiterite flotation for decades. The earliest reports on cassiterite flotation date back to the 1930s, when fatty acid collectors were used. After nearly a century of development, cassiterite flotation has been extensively studied and applied, and the range of cassiterite collectors has significantly expanded.

### 3.1. Fatty Acid Collectors

**Figure 5:**
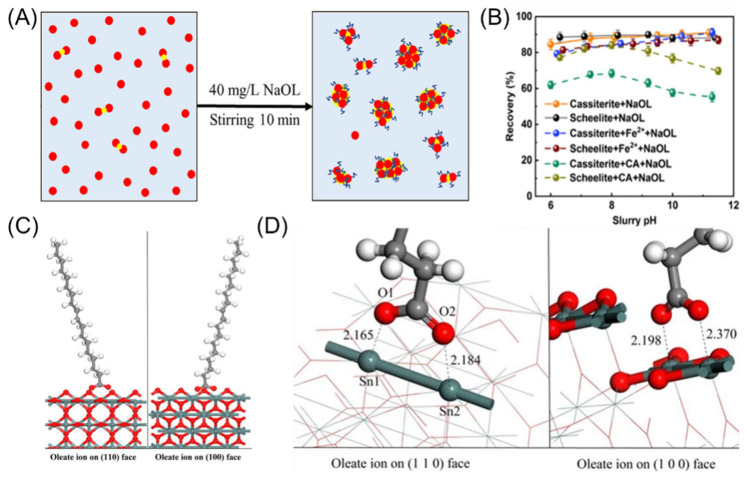
(**A**,**B**) Schematic diagram and selectivity of sodium oleate adsorption [65,66]. (**C**,**D**) Binding details of oleate anions on cassiterite (1 1 0) and (1 0 0) surfaces [68].

The first collectors used in cassiterite flotation were fatty acid-based collectors, such as oleic acid and dicarboxylic acids [63,64]. Oleic acid is a highly effective collector, capable of collecting various minerals, including cassiterite. However, this broad collector ability leads to poor selectivity (as shown in Figure 5A,B) [65,66]. In cassiterite flotation, the use of fatty acid collectors requires a large number of depressants during the flotation process and often necessitates higher flotation temperatures to achieve effective separation [67]. Additionally, fatty acid collectors have poor solubility; however, this issue has been addressed through modifications such as emulsification, sulfonation, halogenation, and sulfation [67]. Fatty acids have the general formula R-COOH, consisting of polar hydrophilic and non-polar hydrophobic groups. The polar hydrophilic group adsorbs onto the mineral surface through bonding interactions, while the non-polar hydrophobic group orients away from the surface, forming a hydrophobic layer. The mechanism of interaction between oleic acid and the cassiterite surface involves the two oxygen atoms in the carboxyl group of oleic acid forming chemical adsorption by bonding with the exposed Sn atoms on the mineral surface (as shown in Figure 5C,D) [64,68]. Studies have found that oleate ions exert the strongest forces on the cassiterite surface, capable of displacing adsorbed H_2_O and OH^−^. During the adsorption process, charge transfer occurs between the atoms [64,68,69]. Therefore, it can be concluded that the adsorption of oleic acid on the cassiterite surface is a chemical adsorption process accompanied by a substitution reaction [69].

Zheng [70] investigated the effect of different pH levels on the flotation efficiency of sodium oleate for cassiterite and found that sodium oleate was most effective at a pH of 9, with a recovery rate reaching approximately 90%. Sodium oleate can bind with various metal ions, leading to its poor selectivity, and it is susceptible to Ca^2+^ and Mg^2+^ ions [71]. Feng [72] studied the effect of calcium ions on sodium oleate’s adsorption and flotation performance on cassiterite and quartz surfaces. Under the influence of Ca^2+^, at a pH of 8.2, the recovery rate difference between cassiterite and quartz in flotation with sodium oleate decreased from 90% to around 70%. For other fatty acid collectors, Baldauf [63] studied the use of alkyl dicarboxylic acids in cassiterite flotation. The results indicated that alkyl dicarboxylic acids have a good collecting ability for coarse cassiterite particles, with the advantage of requiring a lower dosage compared to sodium oleate. Cao [73] studied ricinoleic acid (RA) as a collector for cassiterite flotation from ores. At a pH of 8, RA proved to be an effective collector for SnO_2_ flotation. The flotation of SnO_2_ is dependent on the adsorption of RA anions onto the Sn sites on the SnO_2_ surface.

### 3.2. Phosphonic Acid Collectors

**Figure 6:**
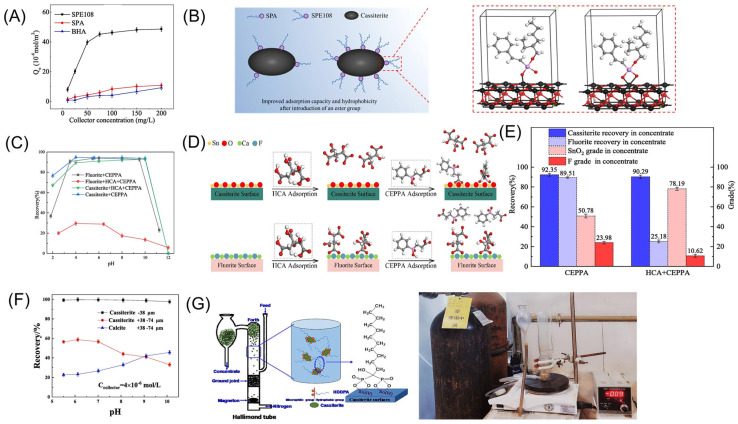
(**A**) Relationship between adsorption capacity of SPE108, SPA, and BHA on cassiterite and dosage [78]. (**B**) Model of interaction between SPE108 and cassiterite [78]. (**C**–**E**) Separation effect and model of HCA + CEPPA on cassiterite and fluorite [80]. (**F**) Relationship between pH and recovery rate in HDDPA separation of cassiterite and calcite [82]. (**G**) Model of interaction between HDDPA and cassiterite [82].

Phosphonic acids are excellent collectors for cassiterite, encompassing aromatic phosphonic acids, aliphatic phosphonic acids, and bisphosphonic acids [19,34]. The advent of these collectors has significantly advanced cassiterite flotation technology. The optimal pH range for cassiterite flotation using phosphonic acid collectors is between 2 and 3. When the pH falls below 2, the recovery rate of cassiterite drops sharply. Within the pH range of 3 to 7, its floatability gradually decreases, but once it exceeds 7, its floatability deteriorates rapidly [74]. Wottgen [53] found that alkanoyl phosphonic acids with carbon chain lengths between C5 and C7 exhibit superior performance in cassiterite flotation. Collins [75] discovered that aromatic phosphonic acids demonstrate significantly greater flotation selectivity than alkanoyl phosphonic acids. One of the most important phosphonic acid collectors for cassiterite is styrene phosphonic acid (SPA). Research indicates that SPA is the easiest to synthesize and the most efficient among phosphonic acid-based cassiterite collectors [76,77]. Gruner [77] conducted a comparative study on the performance of SPA, Asparal-F (a salt of succinamide acid), C12-C16 alkyl hydroxamic acids, bisphosphonic acids (Flotol-7,9), Aeropromoter-845, Alcopol-CA-540, and benzyl arsenic acid in cassiterite flotation. The results demonstrated that only using SPA achieved a Sn concentrate with a grade exceeding 40% and a high recovery rate. Huang [78] was the first to introduce the novel surfactant, styrene phosphonic acid mono-iso-octyl ester (SPE108), as a cassiterite flotation collector. SPE108 demonstrated a stronger electron-donating ability than SPA and BHA (Figure 6A). It was proposed that SPE108 primarily achieves cassiterite adsorption through the cleavage of the PO-H bond and the formation of a PO-Sn bond (Figure 6B). Gong [79] applied 2-carboxyethyl phenyl phosphonic acid (CEPPA) in cassiterite flotation, finding that CEPPA exhibits strong collecting ability for both cassiterite and fluorite across a wide pH range. In contrast, its collecting ability for quartz is relatively weak. The four oxygen atoms of CEPPA react with the Sn atoms on the cassiterite surface, forming a complex similar to a Sn-CEPPA precipitate. Hydrogen bond adsorption may also occur between the terminal hydroxyl groups and the CEPPA anions. Zhu [80] improved the flotation separation of cassiterite and fluorite by introducing hydroxycitric acid (HCA) as a fluorite depressant and CEPPA as a collector. This approach achieved a cassiterite concentrate grade of 78.19% and a recovery rate of 90.29% (Figure 6C–E). Tan [81] studied the flotation behavior of (1-hydroxy-2-methyl-2-octenyl) phosphonic acid (HEPA) on cassiterite. At a concentration of 50 mg/L HEPA, the recovery rate of cassiterite remained above 90% across a broad pH range of 2 to 9. Zeta potential measurements and FTIR analysis indicated that the adsorption of HEPA on cassiterite is primarily attributed to chemisorption between the HEPA anion and the Sn species on the mineral surface. Fan [82] enhanced cassiterite flotation using 1-hydroxy dodecene-1,1-diphosphonic acid (HDDPA). Cassiterite exhibited good floatability in the pH range of 5.5 to 8.0, with HDDPA achieving efficient separation of cassiterite from calcite at around 6.0 (Figure 6F). Mechanism analysis revealed that the Sn^4+^ on the cassiterite surface interacts with the oxygen atom of the OP(=O)-O group in HDDPA, forming a four-membered ring structure that imparts hydrophobic properties to the cassiterite (Figure 6G). Phosphonic acid-based collectors have gradually replaced arsenic acid collectors due to their excellent collecting ability and non-toxicity or low toxicity. However, they face several challenges in industrial applications due to their high cost, susceptibility to metal ion interference, and the significant environmental pollution associated with traditional synthesis methods using phosphorous halide reagents.

### 3.3. Sulphosuccinamate Collectors

**Figure 7:**
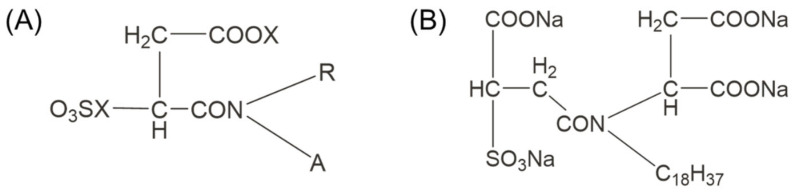
(**A**) General formula of sulphosuccinamate. (**B**) Aerosol-22 molecular formula.

Sulphosuccinamates are amphoteric surfactants that typically contain one or more carboxyl groups in addition to the sulfonic acid group (Figure 7A) [83]. These agents are cost-effective, low in toxicity, and biodegradable, which has led to their widespread use globally. A notable example is Aerosol-22 (Figure 7B), synthesized by the Kunming Institute of Metallurgy (China) [84,85]. The flotation of cassiterite using sulphosuccinamate occurs under conditions with a pH value below 2.5, at which the cassiterite surface is positively charged. Once the pH exceeds cassiterite’s IEP, cassiterite’s recovery rate rapidly declines [86]. Choi [87] discovered that the action of sulphosuccinamate on the surface of cassiterite is a form of chemical adsorption, wherein the atoms in the carboxyl and sulfonic acid groups of the sulphosuccinamate can bond with the Sn atoms on the cassiterite surface, forming various chelate ring adsorption structures. Although sulphosuccinamate exhibits excellent performance, it suffers from drawbacks such as poor selectivity and the necessity for use under strongly acidic conditions. As cassiterite grades decrease and associated resources become more complex, these collectors require the concomitant use of modifiers and depressants for optimal effectiveness.

### 3.4. Hydroxamic Acid Collectors

**Figure 8:**
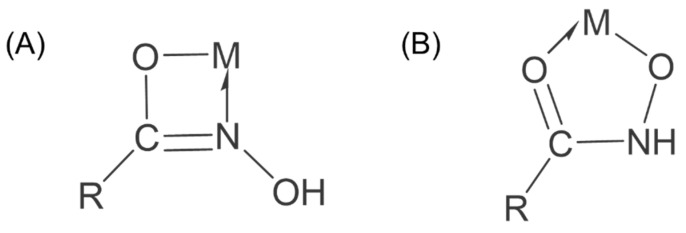
Tetradentate (**A**) and pentadentate (**B**) ring forms of hydroxamic acids.

Hydroxamic acids are chelating collectors in two isomeric forms: oxhydroxamic acid and isohydroxamic acid, with isohydroxamic acid being the predominant form. The chemical structure of hydroxamic acids consists of a non-polar group (R-) and a polar (-COONH) group [88]. The oxygen and nitrogen atoms in the polar group of hydroxamic acids each possess a lone pair of electrons, making them prone to form cyclic metal ion complexes with metal ions. While the tetradentate chelate complexes formed with metal ions are less stable than pentagonal ring chelates, hydroxamic acids favor forming five-membered ring chelates [89,90]. The tetradentate and pentadentate ring forms of hydroxamic acids are shown in Figure 8.

**Figure 9:**
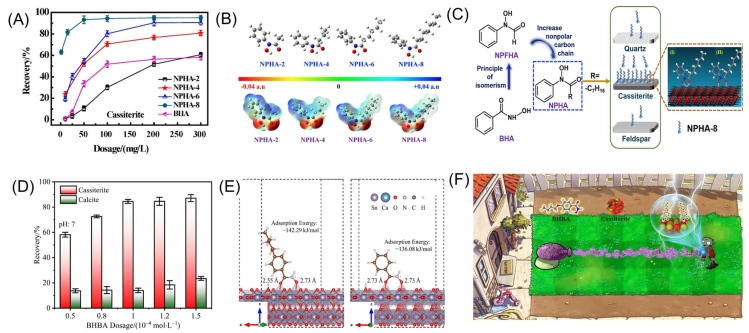
(**A**–**C**) Flotation effect and mechanism analysis of NHPA [97]. (**D**–**F**) Flotation effect and mechanism analysis of BHBA [98].

Familiar hydroxamic acid-based collectors include octylhydroxamic acid (OHA), salicylhydroxamic acid (SHA), and benzohydroxamic acid (BHA). The mechanism of action involves the polar groups in the hydroxamic acid forming chelate rings on the surface of cassiterite, primarily through chemical adsorption, accompanied by physical adsorption [91,92]. Aromatic hydrocarbon-based hydroxamic acids, such as SHA and BHA, require activation by Pb^2+^ to exhibit enhanced selectivity and collecting ability [93,94,95]. The flotation behavior of hydrocarbon-based hydroxamic acids is closely related to the length of the carbon chain. Hydrocarbon hydroxamic acid salts with longer carbon chains exhibit smaller energy gaps and larger dipole moments, making them more prone to adsorption on the SnO_2_ surface through chelate ring formation. An appropriate increase in carbon chain length enhances the collecting ability of hydrocarbon hydroxamic acid salts on cassiterite under acidic to weakly alkaline conditions [96]. Lu [97] synthesized N-phenylacetylisohydroxamic acid (NPHA) by modifying the structure of BHA. As shown in Figure 9A, the effect of the carbon chain length on the flotation performance was investigated. The results indicated that NPHA-6 and NPHA-8 exhibit superior flotation performance on cassiterite compared to BHA, effectively separating cassiterite, quartz, and feldspar over a wide pH range. Both form five-membered chelate rings on the mineral surface through chemical adsorption (Figure 9B,C). Recently, collectors derived from hydroxamic acid with functional group modifications have been progressively applied in cassiterite flotation. Ren [98] proposed the introduction of a butoxy group into the molecular structure of BHA to synthesize 4-butoxy-N-hydroxybenzamide (BHBA). When used as a collector in the flotation separation of cassiterite and calcite, BHBA maintained the selectivity of BHA while enhancing its collecting ability (Figure 9D). This improvement was attributed to its longer hydrophobic carbon chain and the electron-donating properties of the butoxy group (Figure 9E,F). Wu [99] investigated the performance of 2-hydroxy-3-naphthylmethylisohydroxamic acid (NHA) in cassiterite flotation. In the presence of NHA, the maximum recovery of cassiterite reached 91.76%. The hydroxyl groups of NHA chemically coordinate with the Sn-OH groups on the cassiterite surface, forming a six-membered chelate ring. This mechanism can be extended to systems involving the interaction of most metal ions with hydroxyl group-containing isohydroxamic acids, which helps to further understand the role of isohydroxamic acid collectors in cassiterite flotation.

**Figure 10:**
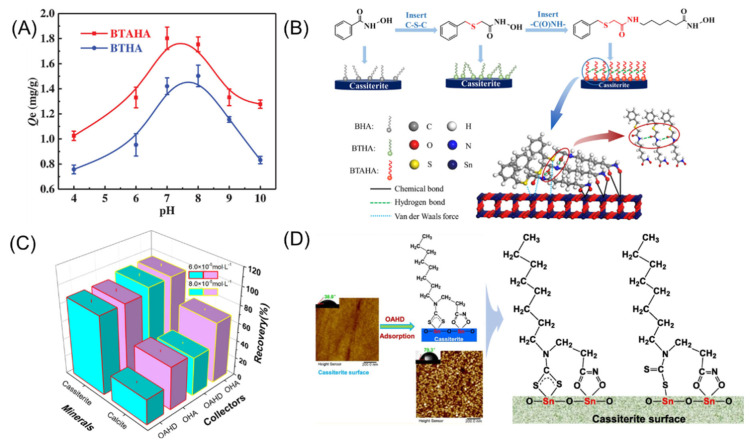
(**A**,**B**) Flotation effect and mechanism analysis of BTAHA and BTHA [100]. (**C**,**D**) Flotation effect and mechanism analysis of OAHD [102].

Enhancing the selectivity of hydroxamic acid-based collectors is a primary research focus. Hydrocarbon-based hydroxamic acids exhibit good collecting ability, but their main drawback is poor selectivity. Sun [100] proposed enhancing the self-assembly and adsorption of sulfur-containing ether hydroxamic acids on the cassiterite surface through amide groups. He [101] synthesized two novel collectors: 2-(benzylthio)-acetohydroxamic acid (BTHA) and *N*-(6-(hydroxyamino)-6-oxhexyl) benzylthioacetamide (BTAHA). By modifying the molecular structure of sulfur-containing ether hydroxamic acids, these compounds’ activity and dispersibility in water were enhanced, facilitating a stronger interaction between the collector and the mineral. The flotation results and mechanistic model are shown in Figure 10A,B. Compared to BTHA, BTAHA exhibits stronger bubble stability, making it more readily adsorbed onto the mineral surface and promoting hydrophobic flotation. Qi [102] synthesized a novel collector, N-[(3-hydroxyamino)-propoxy]-*N*-octyldithiocarbamate (OAHD), by adding a sulfur-containing side chain at the same end, forming both -NC(=S)S- and -C(=O)NHOH bifunctional groups. The sulfur-containing side chain demonstrated differential adsorption, showing adsorption on the cassiterite surface but no adsorption on the calcite surface, thereby enhancing the surface hydrophilicity of calcite and achieving effective selective enrichment of cassiterite (Figure 10C,D). Studies have shown that increasing the number of hydroxamic groups can separate the target mineral from gangue minerals [103,104]. Aromatic hydrocarbon-based hydroxamic acids exhibit good selectivity but have poor hydrophobicity. By adding oxygen-containing substituents (-OCH_3_) or hydrophobic chains to the benzene ring of benzohydroxamic acid, the flotationability of the mineral can also be enhanced. Thus, Zhao [105] synthesized p-methoxybenzohydroxamic acid (PMOB) as a novel collector to improve the recovery of cassiterite in flotation. The recovery rate of cassiterite using PMOB as a collector was 90.08%, compared to a recovery rate of 84.67% using BHA. The -OCH_3_ substituent imparts to PMOB a stronger electron-donating ability and hydrophobicity compared to BHA, p-methylbenzohydroxamic acid (PMB), and p-hydroxybenzohydroxamic acid (PHB), thereby enhancing its flotation performance. In summary, hydroxamic acid-based collectors form stable chelate rings with cassiterite, offering advantages such as high selectivity, strong collecting ability, and environmental friendliness, demonstrating significant potential for development in flotation practices.

### 3.5. Combined Collectors

**Figure 11:**
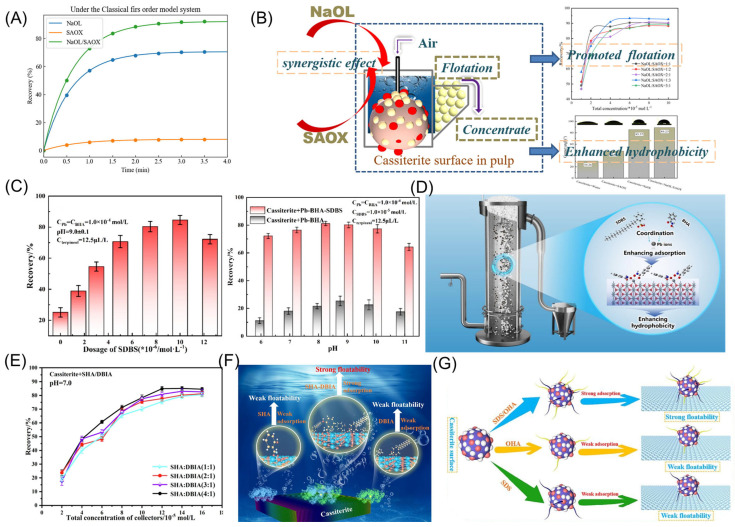
(**A**,**B**) Flotation effect and cassiterite flotation model of NaOL/SAOX [112]. (**C**,**D**) Flotation effect and cassiterite flotation model of Pb-BHA/SDBS [113]. (**E**,**F**) Flotation effect and cassiterite flotation model of SHA/DBIA [115]. (**G**) Cassiterite flotation model of SDS/OHA [116].

Single flotation reagents are increasingly inadequate to meet the production needs of mining enterprises [106]. Combined reagents are often used in flotation processes to enhance the adaptability of flotation reagents for cassiterite flotation [107,108]. Combined collectors typically combine two or more cassiterite flotation collectors, allowing for a balance between collecting ability and selectivity, optimizing the collector’s performance in cassiterite separation [109,110,111]. Huang [112] proposed a combined collector for cassiterite flotation containing sodium oleate (NaOL) and salicylaldoxime (SAOX). At a total concentration of 0.6 × 10^−4^ mol/L and a molar ratio of NaOL to SAOX of 1:3, the cassiterite recovery rate reached 92.00% (Figure 11A,B). Fu [113] introduced a new ligand, sodium dodecylbenzenesulfonate (SDBS), into the structure of the lead-benzohydroxamic acid complex (Pb-BHA) to form a combined collector. This approach increased the flotation recovery of cassiterite from 30% to 80% (Figure 11C). The adsorption of Pb-BHA-SDBS on the cassiterite surface formed large colloidal aggregates, further enhancing cassiterite’s surface roughness and hydrophobicity, thereby improving its flotation performance (Figure 11D). Miao [114] studied the self-assembly of phosphoric acid (SPA) and dodecylbenzenesulfonic acid isopropylamine (DBIA) as a combined collector for cassiterite flotation. The results showed that the SPA/DBIA combined collector achieved a cassiterite flotation recovery rate of 80.88% at a concentration of 1.2 × 10^−4^ mol/L. The addition of SPA caused DBIA to continuously adsorb onto the cassiterite surface, forming a more stable and compact adsorption layer (Figure 11A). Building on this, they further studied the self-assembly of SHA and DBIA. When used individually, cassiterite’s maximum flotation recovery rates were 45.73% for SHA and 71.25% for DBIA [115]. In contrast, the flotation recovery rate using the developed combined surfactant at the same reagent concentration was 84.92%. The hydrophobic carbon chain of the SHA/DBIA self-assembled surfactant eliminates the polar aqueous environment, leading to spontaneous self-assembly in the solution (Figure 11E,F). This significantly reduces the surface tension of the solution and enhances its surface activity. Miao [116] combined OHA with sodium dodecyl sulfate (SDS), and AFM analysis revealed that the combined collector exhibited both dot-like and layered adsorption on the cassiterite surface (Figure 11G). This indicated co-adsorption of the combined collectors on the mineral surface, resulting in a cassiterite flotation recovery rate exceeding 90%. The combination of collectors exhibits a synergistic effect, where their performance complements each other for greater efficiency, reduces reagent consumption, and makes the process more economical. The combined agents are also less toxic, making them more environmentally friendly. Therefore, combined collectors are an essential approach for addressing the recovery of challenging cassiterite.

### 3.6. Other Collectors

In the late 1950s, with P-tolyl arsonic acid’s application in fine cassiterite flotation, arsenic acid-based collectors were officially introduced into flotation processes [63]. Arsenic acid-based collectors primarily encompass two major categories: aromatic arsenic acids and aliphatic arsenic acids. These collectors are derived from arsenic acid, AsO(OH)_3_, through the substitution of hydroxyl groups [117]. Arsenic acid-based collectors can react with Sn in a mildly acidic slurry, forming a white precipitate. Zhang [118] demonstrated that the adsorption capacity of toluene arsenic acid on the cassiterite surface reaches its peak at a pH of approximately 4. The adsorption mechanism is primarily chemisorption, supplemented by electrostatic interactions, hydrogen bonding, and molecular adsorption. Arsenic acid-based collectors exhibit exceptional selectivity and are minimally affected by Ca^2+^ and Mg^2+^, making them ideal collectors for cassiterite. However, their high cost, toxicity, and significant environmental impact have severely constrained their practical application under increasing environmental regulations. Biological studies have shown that specific microbial cells or their metabolic products can interact with mineral surfaces, altering their properties and affecting the mineral’s floatability. As a result, these microbial substances can be used as flotation reagents [119,120,121]. Gonzales [122] studied the application of *Rhodobacter sphaeroides* as a microbial collector in the electrochemical flotation of fine cassiterite particles. The interaction between the microbial cells and the cassiterite surface was analyzed using zeta potential measurement, contact angle testing, and adsorption analysis. The study found that the surface of *Rhodobacter sphaeroides* exhibited hydrophobicity, and after the microbial cells interacted with the cassiterite surface, the contact angle of the cassiterite surface increased. In the flotation experiments, under the conditions of pH = 5, bacterial concentration of 50 mg/L, and a current density of 51.4 mA/cm^2^, the flotation recovery rate of cassiterite was approximately 64.5%. Collectors with dual mineralophilic groups have been shown to enhance the recovery efficiency of cassiterite [123,124]. For example, *S*-((2-hydroxyamino)-2-acetyl)-*O*-octyl-dithiocarbamate (HAOODE) contains two functional groups (dithiocarbamate and hydroxamic acid groups), in contrast to the conventional collector OHA [125]. The metal–HAOODE complexes exhibit enhanced hydrophobicity and greater stability, featuring the hydroxamic acid salt-(O, O)-metal and -O-C(-S-metal)-S- configurations. As a result, they demonstrate stronger chemical adsorption on metal oxide ores. Although research on the flotation separation of cassiterite from gangue minerals using HAOODE has not yet been reported, satisfactory flotation results have been achieved in separating malachite from calcite or malachite from quartz. This suggests that HAOODE holds potential for application in cassiterite flotation, with the possibility of necessary modifications in the future. Other types of collectors have also been applied in cassiterite flotation, such as cationic collectors, alkyl sulfonic (sulfuric) acids, and non-ionic collectors. Dodecylamine and octadecylamine are cationic collectors that improve cassiterite floatability through electrostatic adsorption and the exchange of hydrogen ions. However, their selectivity is slightly lower in separating complex ores [61,126,127]. Alkyl sulfonic (sulfuric) acid collectors, such as sodium dodecylbenzenesulfonate, possess collecting and foaming properties. However, their selectivity for cassiterite is generally moderate, and they are sensitive to Ca^2+^ and Fe^3+^. Therefore, further modification of the reagent is necessary to achieve better performance [128]. Non-ionic collectors, such as octylphenol ethoxylates and octylphenol polyoxyethylene ethers, interact with cassiterite particles through hydrogen bonding and van der Waals forces. They are less sensitive to Ca^2+^ and heavy metal ions, making them a more stable option in complex ore flotation [111].

## 4. Depressants

### 4.1. Inorganic Depressants

**Figure 12:**
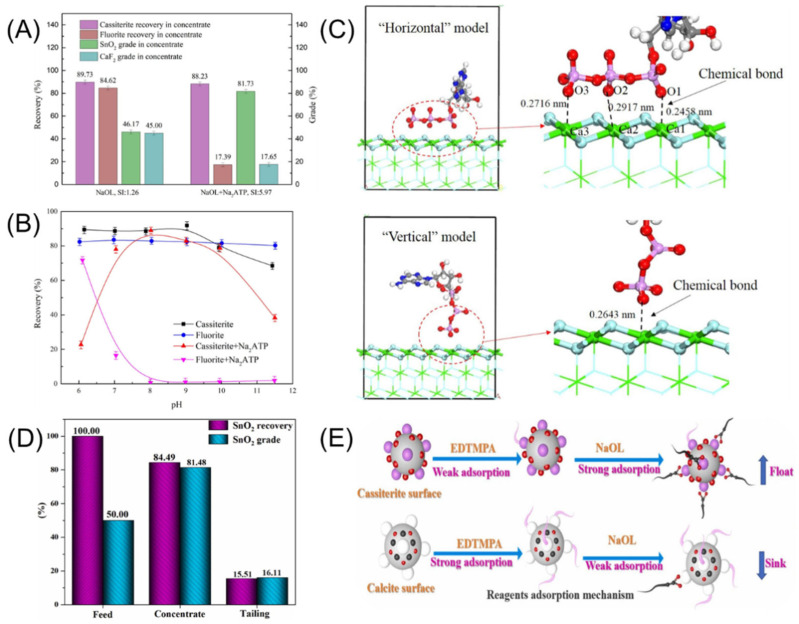
(**A**–**C**) Cassiterite flotation effect and model of Na_2_ATP [142]. (**D**,**E**) Cassiterite flotation model of EDTMPA [143].

Relying solely on collectors is often insufficient to meet the requirements of cassiterite flotation. The synergistic effects between different reagents, along with solvent effects and salting-out effects, can enhance the flotation separation performance [129,130,131,132]. Depressants can improve the hydrophilicity of minerals, thereby suppressing their interaction with collectors. Cassiterite depressants are classified into organic and inorganic types. Common inorganic depressants include water glass, sodium sulfide, and sodium hexametaphosphate [133,134,135]. Water glass is commonly used to depress silicate and silica gangue minerals by forming calcium silicate, precipitating Ca^2+^ ions from the solution [136]. Acidified water glass is most effective in depressing silicate minerals while also playing a role in regulating the pH of the pulp [137]. Sodium fluosilicate is also a commonly used depressant in the flotation of fine cassiterite [138]. Taking the depression of calcite as an example [139], under alkaline conditions, sodium fluosilicate dissociates to produce F^−^, SiO(OH)_3_^−^, and SiF_6_^2−^, which adsorb onto the calcite surface through electrostatic interactions, thereby hindering further adsorption of the collector. Sodium hexametaphosphate and sodium sulfide are also effective depressants for gangue minerals in cassiterite flotation. They can adsorb onto the mineral surfaces, enhancing the steric hindrance effect between mineral particles. These two reagents can also form complexes with metal cations such as Ca^2+^ and Mg^2+^, thereby depressing the flotation of Ca and Mg gangue minerals [140]. Targeting the common Ca-bearing gangue minerals in cassiterite, Wang [141,142] synthesized two eco-friendly phosphonate depressants (PBTCA and Na_2_ATP), which enabled efficient flotation separation of cassiterite from fluorite. The results showed that both depressants exhibited significantly better fluorite adsorption than cassiterite. The phosphonate groups formed chemical adsorption with Ca^2+^ on the fluorite surface, hindering the adsorption of NaOL on the fluorite surface. This enhanced the flotation selectivity of the collector and promoted the efficient separation of cassiterite from fluorite (Figure 12A–C). Miao [143] synthesized (ethylenedinitrilo)-tetramethylenephosphonic acid (EDTMPA) as a calcite depressant. Similar to Na_2_ATP and PBTCA, the phosphonate group exhibits a strong interaction with the Ca^2+^ on the calcite surface while showing weaker adsorption on cassiterite (Figure 12D,E). Depressants also have a certain impact on cassiterite flotation. Therefore, selecting the appropriate reagent regime and considering the combined flotation performance of both collectors and depressants is a key challenge in separating cassiterite from other minerals.

### 4.2. Organic Depressants

**Figure 13:**
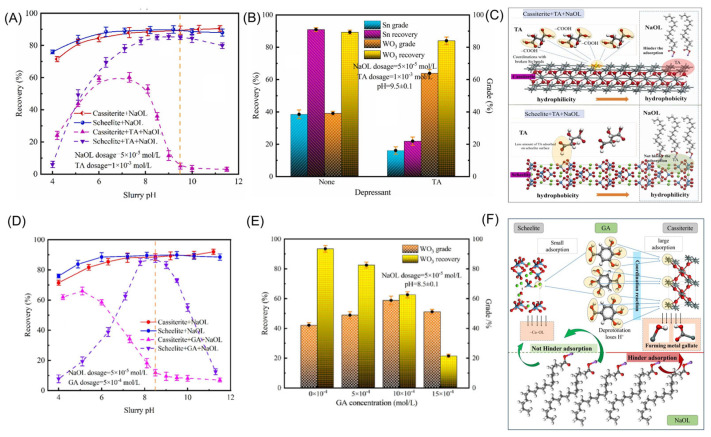
(**A**,**B**) Depression effect of TA on scheelite and cassiterite [152]. (**C**) Cassiterite flotation model of TA [152]. (**D**,**E**) Depression effect of GA on scheelite and cassiterite [153]. (**F**) Cassiterite flotation model of GA [153].

There is a wide variety of organic depressants, and in recent years, researchers have conducted extensive studies on this topic [144,145,146,147]. Carboxymethylcellulose (CMC) is an effective depressant for calcite, capable of working synergistically with various collectors, such as NaOL [148]. When using oleic acid to flotation cassiterite at pH = 8.1, CMC exhibits the most effective depressed performance on calcite [149]. Citric acid also depresses cassiterite. Liu [149] studied the depressed effect of citric acid on cassiterite within the SHA system. The addition of citric acid causes the surface potential of cassiterite to shift in a negative direction. Its carboxyl groups react with the Sn atoms on the cassiterite surface, enhancing the hydrophilicity of the cassiterite surface. At the same time, it weakens the adsorption of salicylic hydroxamic acid on the cassiterite surface, thereby deteriorating the flotation of cassiterite. Lv [150] used SPA as a collector and galactomannan (GM) as a depressant. Under conditions of pH 8.00, GM dosage of 1.0 mg/L, and SPA dosage of 80 mg/L, it was found that GM exerted a strong depressed effect on the flotation of cassiterite. The adsorption density of GM on cassiterite is much higher than on fluorite. GM increases cassiterite’s hydrophilicity while hindering SPA’s adsorption on the cassiterite surface. Furthermore, XPS results indicate that the Sn atom is the primary active site for the reaction between cassiterite and GM. As a result, GM can selectively depress cassiterite flotation with minimal impact on fluorite flotation. Yao [151] used pectin extracted from citrus peels as a depressant for calcite. When the pectin dosage was 10 mg/L, the recovery rate of cassiterite reached 80%. Citrus pectin offers advantages such as low cost, high selectivity, and environmental suitability, making it a promising alternative to traditional reagents. As scheelite and cassiterite occur as associated minerals, achieving their separation is also crucial. Zheng [152] selected an environmentally friendly depressant, DL-tartaric acid(TA), to achieve the selective flotation separation of scheelite and cassiterite. In the flotation concentrate, the WO_3_ and Sn grades were 63.88% and 16.12%, with recoveries of 84.03% and 21.91%, respectively (Figure 13A,B). The polar carboxyl group, composed of C-O and C=O bonds, in the depressant undergoes a coordination reaction with the Sn active sites on the cassiterite surface, resulting in strong chemical adsorption of the depressant on the cassiterite surface (Figure 13C). Zheng [153] further proposed the selective depression mechanism of gallic acid (GA) in the flotation separation of scheelite and cassiterite. Similar to TA, GA adsorbs onto the mineral surface through its -COOH and -OH groups, forming metal gallates. This interaction creates a textured oxidation layer enriched with hydroxyl groups, reducing the hydrophobicity of the cassiterite surface and thereby depressing its flotation (Figure 13D–F). Zheng [154] proposed soluble starch (SS) as an effective depressant for the flotation separation of scheelite and cassiterite. The polymeric organic depressant SS contains abundant hydrophilic groups, enabling it to stably adsorb onto the Sn active sites on the cassiterite surface, thereby hindering the collector’s NaOL adsorption. The dosage and cost of depressants during flotation are also key factors. Although certain organic depressants show promising results in laboratory settings, practical applications may face challenges such as high costs or difficulties in large-scale production. Therefore, developing green, environmentally friendly, efficient, and cost-effective organic depressants represents a significant challenge for future research.

### 4.3. Combined Depressants

**Figure 14:**
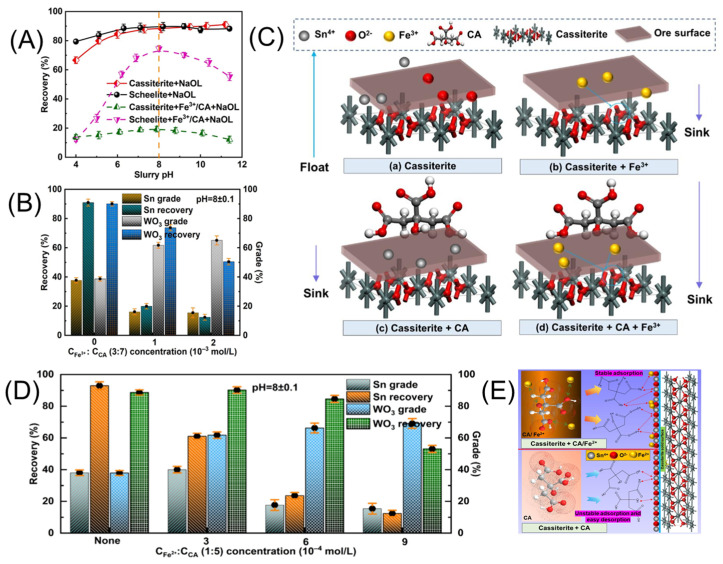
Fe^3+^/CA cassiterite flotation effect (**A**,**B**) and model (**C**) [161]. Fe^2+^/CA cassiterite flotation effect (**D**) and model (**E**) [66].

The combined use of depressants can fully harness their synergistic depressed effects, achieving efficient flotation separation [155,156]. Combined depressants are typically a mixture of several inorganic or organic depressants used to suppress various gangue minerals, thereby achieving efficient separation of complex ores. For instance, combining sodium hexametaphosphate with larch tannin, sodium carbonate with lignin, and sodium metasilicate with CMC fully exploits the synergistic depressed effects of both inorganic and organic components [157]. Zhao [158] studied the impact of the metal–inorganic complex depressant ALSS, composed of aluminum sulfate and water glass, on the flotation separation of cassiterite and the typical gangue mineral calcite. The study found that the depressant ALSS tends to adsorb more strongly onto the calcite surface, reducing the adsorption of the collector, NaOL, and simultaneously decreasing the floatability of calcite, thereby achieving selective adsorption on calcite. The combined use of metal ions and depressants can also yield excellent results. For example, combining metal ions and water glass as a combined depressant is more effective in suppressing quartz than using a single depressant [159,160]. Zheng [161] investigated the selective depressant behavior and mechanism of a combined depressant consisting of Fe^3+^ and citric acid on cassiterite. Experimental studies on single minerals and artificially mixed ores revealed that individual FeCl_3_ or citric acid exhibited weak depressed capabilities, with poor selectivity for cassiterite. However, combining FeCl_3_ and citric acid in a 3:7 mass ratio achieved better separation results (Figure 14A–C). Zheng [66] also explored the combination of Fe^2+^/CA for separating scheelite and cassiterite. Fe^2+^/CA exhibited a high adsorption affinity for the cassiterite surface, primarily composed of L^3−^, Fe^2+^, and FeOH^+^ components (Figure 14D). Additionally, the FeOH^+^ complex formed by the pre-reaction between Fe^2+^ and CA exhibits significant chemical adsorption at the Sn active sites, thereby depressing cassiterite (Figure 14E). The choice of depressant should be based on the specific type of gangue mineral. However, during the flotation process, depressants may also exert some degree of depression on the target mineral. The interactions between multiple minerals and various reagents in the flotation system increase the research complexity, especially under conditions where the slurry composition is complex, the depressant dosage is low, yet high efficiency is required. Under such circumstances, studying the independent effects of a single depressant becomes challenging. Balancing performance and cost-effectiveness is a key challenge in practical applications.

## 5. Activators

**Figure 15:**
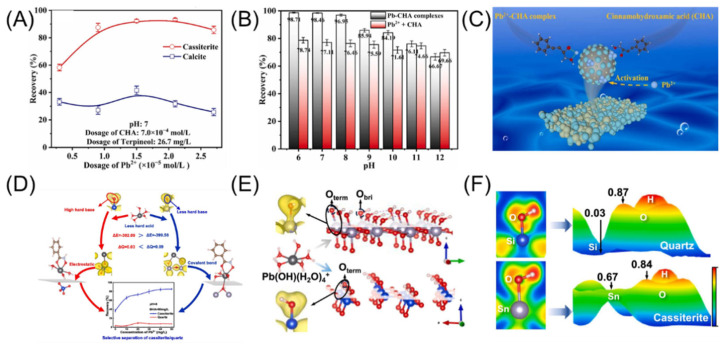
(**A**–**C**) Cassiterite flotation effect and model of Pb^2+^-CHA [167]. (**D**–**F**) Model for selective activation mechanism analysis of cassiterite in quartz by Pb^2+^ [168].

As high-quality tin ore resources continue to be depleted, the associated components in tin ores become more complex. Therefore, relying solely on a single flotation collector is insufficient to meet the separation requirements of cassiterite from other minerals. It is necessary to introduce suitable activators to optimize the slurry environment and achieve efficient cassiterite separation [162]. Cassiterite activators are typically metal cations, which adsorb onto the cassiterite surface, increasing the active sites for collector interaction. The combination of Pb^2+^ with collectors has been applied in the flotation of various ores and shows significant potential. The combination of Pb^2+^ with collectors has been applied in the flotation of various ores, demonstrating considerable potential [163,164]. In the cassiterite flotation system, SHA and cinnamic hydroxamic acid are influenced by Pb^2+^, and compared to the absence of Pb^2+^, the floatability of cassiterite is enhanced in both cases. The activation effect of Pb^2+^ and Pb(OH)^+^ increases the number of active sites on the cassiterite surface, thereby improving the flotation recovery rate [165]. The method of adding Pb^2+^ also affects the flotation performance. Tian [166] studied the effect of Pb^2+^ on cassiterite flotation using BHA. The results indicated that the sorting performance of Pb^2+^-BHA was better than when Pb^2+^ and BHA were added sequentially in the same dosage. Nie [167] employed a flotation system composed of CHA collector and Pb^2+^ activator, achieving a cassiterite recovery rate of 90% (Figure 15A,B). The activation mechanism is attributed to forming Pb-CHA complexes (Figure 15C). Miao [168] used the selective flotation of cassiterite from quartz activated by Pb^2+^ as an example to illustrate the crucial role of surface hydroxyl groups in selective flotation. The interaction between metal ions and mineral surface hydroxyl groups must adhere to specific hardness and softness principles. The hardness of hydroxyl or oxygen atoms varies on different mineral surfaces, primarily due to the different chemical bonds between the central atoms and the oxygen atoms (Figure 15D–F). The application of the hard and soft acid–base (HSAB) principle can explain the selective adsorption of metal ions on different mineral surfaces. This understanding is crucial for selecting appropriate activating ions or collectors.

**Figure 16:**
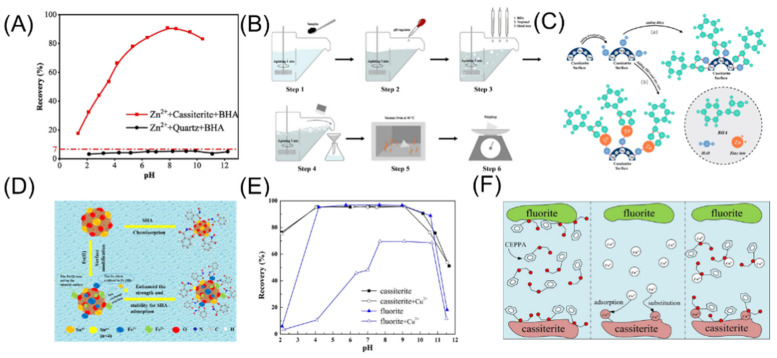
(**A**) Cassiterite flotation effect and model of Zn^2+^-BHA [170]. (**B**,**C**) Cassiterite flotation model of Fe^2+^-SHA [171]. (**D**–**F**) Cassiterite flotation effect and model of Cu^2+^-CEPPA [172].

Due to the potential environmental pollution caused by Pb^2+^, many researchers have explored alternative ions to replace Pb^2+^. Tian [169] investigated the effect of Fe^3+^ as an activator on the flotation performance of cassiterite. Replacing Pb^2+^ with Fe^3+^ can reduce environmental pollution. In the presence of Fe^3+^, the adsorption of BHA on the cassiterite surface significantly increased. Cao [170] replaced Pb^2+^ with Zn^2+^ to study the effect on cassiterite flotation using BHA. The results showed that Zn^2+^ has a more substantial activation effect on cassiterite than Pb^2+^. In the presence of Zn^2+^, the maximum recovery rate of cassiterite reached 90.54% (Figure 16A,B). Zn forms Zn-O bonds on the cassiterite surface, and these bonds combine with BHA molecules to create a new chelate ring, enhancing adsorption capacity and improving cassiterite recovery (Figure 16C). Cao [171] subsequently investigated the effect of Fe^2+^ on the adsorption of SHA on the cassiterite surface. After activation by Fe^2+^, the adsorption capacity of SHA on the mineral surface was significantly enhanced (Figure 16D). Gong [172] studied the effect of Cu^2+^ on the flotation separation of cassiterite and fluorite using styrene phosphonic acid as a collector. Styrene phosphonic acid demonstrated good collecting ability for cassiterite and fluorite (Figure 16E). Cu^2+^ exhibited a strong depressed effect on fluorite, hindering the adsorption of styrene phosphonic acid on the fluorite surface, but had little impact on the adsorption of styrene phosphonic acid on the cassiterite surface (Figure 16F). Feng [173] investigated the effect of Mg^2+^ and Ca^2+^ on the flotation separation of cassiterite and quartz. The results showed that under acidic conditions, Mg^2+^ and Ca^2+^ did not adsorb onto the surfaces of cassiterite and quartz. At a pH of 8.1, a small amount of Mg^2+^ and Ca^2+^ adsorbed onto the mineral surfaces, reducing the interaction between NaOL and cassiterite particles. Under strongly alkaline conditions, a large amount of Mg^2+^ and Ca^2+^ adsorbed onto the surfaces of both cassiterite and quartz, weakening the adsorption of NaOL on cassiterite while enhancing its adsorption on quartz, thereby increasing the difficulty of flotation separation between the two minerals. In multi-mineral systems, activators often need to selectively act on the target mineral without activating other minerals. However, controlling the selectivity of activators is challenging, especially when the mineral properties are similar, which can lead to over-activation of non-target minerals and reduce separation efficiency. Flotation pulps often contain multiple minerals and ions, which can compete for adsorption or interfere with reactions involving the activators, making their effectiveness unpredictable. Therefore, the interaction mechanisms between activators and mineral surfaces require more in-depth research.

## 6. Conclusions and Future Perspectives

This review consolidates the current advancements in flotation reagents related to cassiterite, providing a detailed summary of their effects on mineral behavior and surface interactions during flotation separation. However, several opportunities and challenges still warrant attention:

(1) To overcome the challenges posed by cassiterite collectors, such as high costs, toxicity, insufficient selectivity, and collecting efficiency, future research should prioritize the computational design of collectors. We propose that the future development of cassiterite flotation reagents should be guided by a “targeted action mechanism”, adopting a rational design approach driven by the synergy of quantum chemistry and molecular simulation. This strategy aims to achieve precise recognition and efficient adsorption of reagents on the cassiterite surface. Compared with traditional empirical methods, this approach can unveil the binding mechanisms between reagent molecules and the electronic structure characteristics of cassiterite at the atomic scale, thereby laying a solid theoretical foundation for the development of highly selective reagents.
(2) Additionally, developing biodegradable collectors derived from natural compounds should be emphasized to minimize environmental impact while preserving flotation performance. We believe that bio-based and biodegradable flotation reagents represent a critical breakthrough for the green transformation of cassiterite flotation. Naturally derived surfactants—such as saponins, flavonoids, tannic acid, and alginates—exhibit excellent biocompatibility and environmental friendliness. Moreover, some of these molecules contain functional groups capable of complexation or hydrophobic interactions with metal mineral surfaces, endowing them with potential collecting or depressing capabilities. In addition, functionalized biopolymers, such as modified chitosan and carboxymethyl cellulose, possess polymer chain structures that enable multipoint adsorption. These materials demonstrate strong mineral surface recognition and controllable release properties, making them promising candidates for the next generation of green flotation reagents.
(3) Exploring the synergistic effects of combining multiple reagents could further enhance selectivity and collecting efficiency. For instance, pairing fatty acids with chelating agents or surfactants could strengthen hydrophobic interactions and reduce reagent consumption. The activation of gangue minerals by metal ions complicates the effective separation of cassiterite from gangue minerals, undermining selectivity. A key strategy for developing selective depressants lies in investigating their electrochemical properties and surface adsorption mechanisms to elucidate their interactions with cassiterite and gangue minerals. Focus should be placed on synthetic polymers and functionalized biopolymers that exhibit selective interactions with gangue minerals, enabling better control over flotation selectivity without inhibiting cassiterite. Integrating depressants with adaptive control systems could dynamically adjust reagent dosages based on real-time monitoring of mineral surface properties, further enhancing separation efficiency.
(4) Metal ions present in flotation pulp can either enhance or hinder cassiterite flotation by altering surface potential and forming complexes that affect mineral selectivity. To optimize the effects of metal ions, reagents that regulate the zeta potential and maintain electrostatic conditions favorable for cassiterite flotation should be developed. Understanding how metal ions influence surface charge can facilitate the design of more efficient flotation environments. Research should also focus on alternative activators, such as rare earth elements or non-toxic chelating agents, to replace traditional pollutant activators like Pb^2+^, thereby enhancing flotation performance while minimizing environmental risks. Additionally, ion-specific reagents can be designed to selectively target harmful ions in the pulp, mitigating their impact on cassiterite flotation without compromising other process parameters.

Advancing the development of novel flotation reagents and control strategies can significantly enhance the flotation efficiency of cassiterite. These innovations not only contribute to the sustainable utilization of tin resources but also promote environmentally friendly mining practices, supporting global efforts in resource conservation and environmental protection.

## Figures and Tables

**Table 1:** Summary of measured IEPs for cassiterite.

Sample Origin	Background Electrolyte	Measurement Technique	IEP	Reference
Australia	-	Electrophoresis	7.3	[51]
The Republic of Zimbabwe	KCl	Streaming potential	3.4	[52]
Congo	KCl	Streaming potential	3.9	[52]
France	KCl	Streaming potential	4.5	[52]
Germany	-	Electrophoresis	5.6	[53]
Bolivia	KCl	potentiometric titration	4	[54]
Canada	KNO_3_	potentiometric titration	5.4	[55]
Germany	-	Electrophoresis	3	[56]
Bolivia	-	Streaming potential	4.5	[57]
Australia	-	Electrophoresis	4.2	[58]
Australia	KCl	Electrophoresis	2.9	[58]

## Data Availability

No new data were created or analyzed in this study.
